# Exploring the Spatial Relative Risk of COVID-19 in Berlin-Neukölln

**DOI:** 10.3390/ijerph20105830

**Published:** 2023-05-16

**Authors:** Christoph Lambio, Tillman Schmitz, Richard Elson, Jeffrey Butler, Alexandra Roth, Silke Feller, Nicolai Savaskan, Tobia Lakes

**Affiliations:** 1 Geography Department, Applied Geoinformation Science Lab, Humboldt-University Berlin, 10099 Berlin, Germany; 2 UK Health Security Agency, 61, Colindale Avenue, London NW9 5EQ, UK; 3 School of Environmental Sciences, University of East Anglia, Norwich Research Park, Norwich NR4 7TJ, UK; 4 Local Health Department Berlin-Neukölln, Gesundheitsamt Neukölln, Blaschkoallee 32, 12359 Berlin, Germany; 5 IRI THESys, Integrative Research Institute on Transformations of Human-Environment Systems, Humboldt-Universität zu Berlin, 10099 Berlin, Germany

**Keywords:** COVID-19, infectious disease, spatial relative risk, kernel density, point data, modifiable areal unit problem

## Abstract

Identifying areas with high and low infection rates can provide important etiological clues. Usually, areas with high and low infection rates are identified by aggregating epidemiological data into geographical units, such as administrative areas. This assumes that the distribution of population numbers, infection rates, and resulting risks is constant across space. This assumption is, however, often false and is commonly known as the modifiable area unit problem. This article develops a spatial relative risk surface by using kernel density estimation to identify statistically significant areas of high risk by comparing the spatial distribution of address-level COVID-19 cases and the underlying population at risk in Berlin-Neukölln. Our findings show that there are varying areas of statistically significant high and low risk that straddle administrative boundaries. The findings of this exploratory analysis further highlight topics such as, e.g., Why were mostly affluent areas affected during the first wave? What lessons can be learned from areas with low infection rates? How important are built structures as drivers of COVID-19? How large is the effect of the socio-economic situation on COVID-19 infections? We conclude that it is of great importance to provide access to and analyse fine-resolution data to be able to understand the spread of the disease and address tailored health measures in urban settings.

## 1. Introduction

On 11 March 2020, the WHO declared the outbreak of the coronavirus disease a global pandemic [1]. Since then, most countries have been experiencing waves of COVID-19 infections, with varying rates of infection over time and space [2]. With the onset of the pandemic, the spatio-temporal distribution of COVID-19 is of particular interest. Areas with high and low infection rates can provide important etiological clues. These clues are crucial to supporting decision-making processes, controlling transmission, properly planning for community actions, and protecting vulnerable groups from infection [3,4,5].

To describe and map the changes in COVID-19 infection rates, many earlier studies throughout the world have often only described the spatio-temporal distribution of COVID-19 infections by aggregating the data to administrative boundaries such as countries, states, districts, or municipalities and displaying the result as choropleth maps, e.g., Arab-Mazar et al. [6], Basnet et al. [7], Guan et al. [8], and Martellucci et al. [9]. Dong et al. [10] developed a dashboard to track the coronavirus disease in real-time, reporting cases at the city, province, and country levels. Yalcin [11] created cartograms at the country level with worldwide coverage for the first 150 days of the pandemic, distorting countries according to their confirmed cases and deaths. In several countries, such as South Korea [4] and Germany [12], an online dashboard was developed where users could, e.g., observe the locations of the patients and identify high-risk locations and infected areas aggregated in administrative areas. Other earlier health science studies do not use administrative areas as aggregational units but aggregate data into regular grids (e.g. [13]).

These geographical units are usually developed for administrative reasons and dissect areas in a way that rarely reflects the complexity of the underlying environment from a health perspective. Most importantly, aggregating data for these areas implicitly assumes that the risk of contracting a disease is constant across these spatial entities. However, this is often not true and was first formally described by Openshaw [14] as the modifiable areal unit problem (MAUP). The MAUP describes the bias when aggregating spatial point data into larger spatial features, i.e., administrative areas or a grid. The resulting summary values from this aggregation depend on the shape and size of the aggregational unit and may mask variations within. For this reason, the resulting values can change when the shape or size of the aggregational unit changes. Analysing spatial point patterns with density-based methods such as kernel density estimation overcomes this problem not by focusing on the distribution superimposed by the aggregational unit [15] but by analysing the intensity and true distribution of the underlying data [16]. Such an estimated density of points at one location can be derived by applying kernel smoothing functions [17] that include the concentration of points within the neighbouring areas, allow for the depiction of smoothed surface maps, and aid in the identification of areas with high or low densities of events, such as identified COVID-19 cases. When it comes to disease mapping, various epidemiological and health science studies have addressed the MAUP throughout the years [18,19,20,21,22,23]. However, only a few studies describe the spatio-temporal pattern of COVID-19 at the point level.

Feitosa et al. [24] analyse point-level data from positive PCR tests to plot the exact locations of infections and deaths with kernel smoothing to create COVID-19 maps for the city of Macaé (Brazil). Sarwar et al. [25] outline several (mostly visual) methods for how geographical information systems (GISs) can be used in control, containment, and precautionary actions in the case of COVID-19. Elson et al. [26] calculated the spatio-temporal relative risk, including surface tolerance contours, highlighting areas of significantly higher risk from confirmed COVID-19 cases in England. Garcia-Morata et al. [15] performed a geo-epidemiological case and control study to estimate the relative risk and its spatial variation of COVID-19 cases in Albacete (Spain) using kernel smoothing techniques to highlight areas of significant incidence and to create a kernel density estimation for a density map. Mohammad Ebrahimi et al. [27] also used kernel smoothing (among other methods) to analyse the spatial intensity of COVID-19 cases in the city of Mashhad, Iran. Xu et al. [28] applied space-time clustering to COVID-19 cases at the national level to identify clusters at the family level, arguing that detecting space-time clusters is of great importance for early warnings and the prevention of future outbreaks.

Even though the studies show promising results and insights into the spatio-temporal characteristics of the COVID-19 pandemic, only a limited number of point-level studies exist, and the lack of high-resolution studies is rarely criticised [29,30]. Additionally, hardly any studies have analysed COVID-19 throughout its complete duration. Often, only a part of the whole pandemic is analysed. This gap in scientific studies might be explained by privacy regulations prohibiting data release at the individual level, the novelty of the pandemic, the missing workflows for data assessment, management, and analysis, and the need for fast research results to support the scientific community involved in COVID-19 research as well as to provide information for policy makers in a global crisis. Nonetheless, the spatio-temporal patterns of COVID-19 on a small scale across the entire duration of the pandemic are most important. This is especially true for heterogeneous urban areas with varying population density, different socio-economic situations, and changing built environments in a small space, which lead to different infectious patterns across space and time. This study addresses this lack of COVID-19 research on a small spatial scale with point data for a local intra-urban setting by developing a relative risk surface, considering the varying spatio-temporal distribution of COVID-19 cases at the address level and the underlying population at risk. The objectives of this study are to (a) describe the spatio-temporal distribution of COVID-19 for the local intra-urban area of Berlin-Neukölln and (b) identify areas of statistically significant relative high and low risk. By choosing a visual-spatial exploratory approach, the results from this analysis provide important etiological clues on hot and cold spots of COVID-19 infections. Moreover, we highlight areas for further research and underline the importance of assessing and analysing detailed health data for adequate health policies.

## 2. Materials and Methods

### 2.1. Study Area

**Figure 1:**
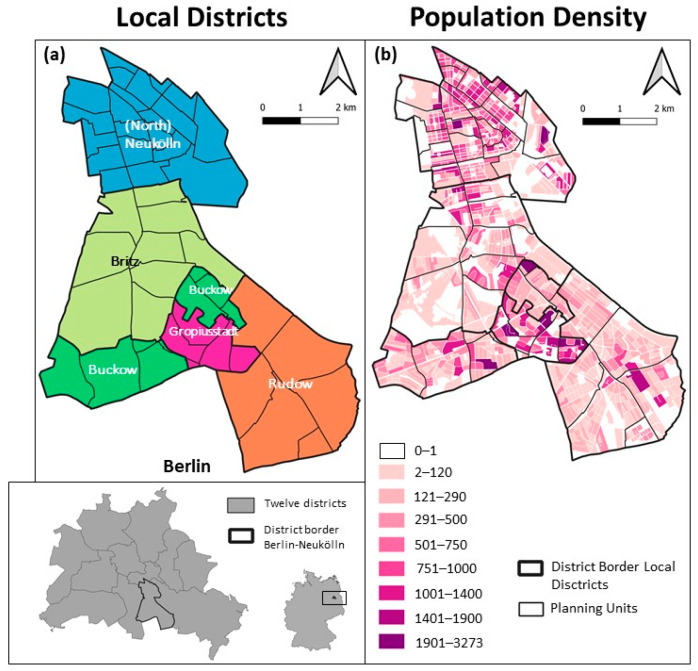

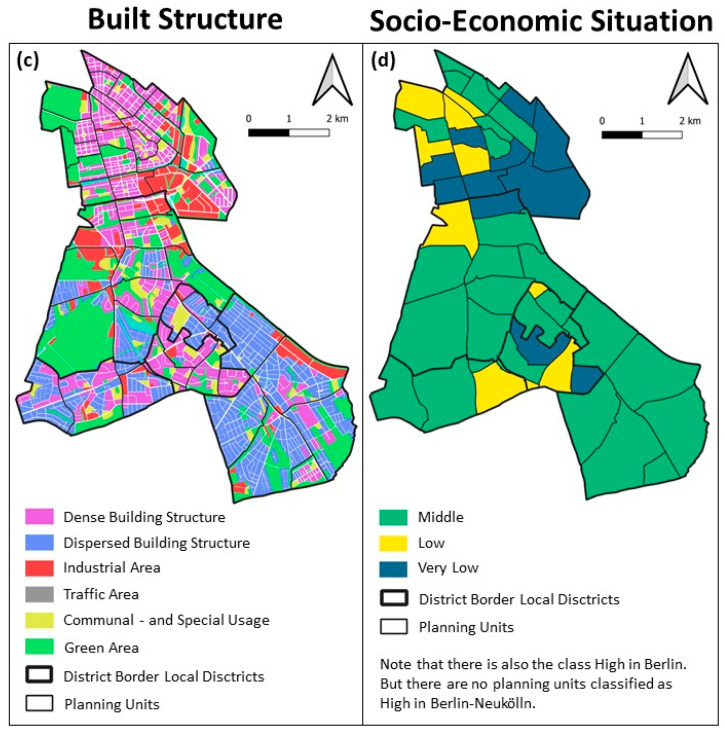
The inset map depicts Berlin and its 12 districts, with Berlin-Neukölln highlighted. (**a**) The five local districts of Berlin-Neukölln and the planning units [40], (**b**) population density of Berlin-Neukölln [41], (**c**) built structure of Berlin-Neukölln [38], and (**d**) socio-economic situation [39]; CRS: 25833.

The study area is Berlin-Neukölln, one of the 12 districts in Berlin. Berlin-Neukölln is a very heterogeneous and diverse district and has often been the subject of (German) social, cultural, and urban studies [31,32,33,34]. There were 327,945 people from 135 different countries living in Berlin-Neukölln in 2020 [35,36]. The district of Berlin-Neukölln has an area of 45 km^2^ and is subdivided into five local districts: Neukölln (from which the district derives its name, also commonly known as North-Neukölln), Britz, Buckow, Rudow, and Gropiusstadt (Figure 1a). Each of these local districts has its own particular socio-economic and environmental characteristics, making Berlin-Neukölln a perfect study site to employ an exploratory approach to COVID-19 data.

Berlin uses a hierarchical, statistical spatial unit system as a basis for the planning and prediction of demographic and socio-economic development: the small-scale lebenswelt-oriented areas [37]. The delineations of the most detailed units (planning units) rely on similar built structures and socio-demographic characteristics to define homogeneous areas within the districts. At the same time, these small-scale lebenswelt-oriented areas are comparable, giving decision-makers as well as scientists a basis for planning, prognosis, and surveillance of socio-economic development in the city of Berlin. In Berlin, there are 542 of these planning units; in Berlin-Neukölln, there are 46 in total (Figure 1a). Nearly all the statistical analyses are performed on these statistical units because most of the available data are aggregated into these aggregational units.

#### 2.1.1. Population

The population density in these local districts varies greatly (Figure 1b, Table 1). The three local districts—(North) Neukölln, Britz, and Rudow—are nearly equal in size, yet four times as many people live in (North) Neukölln as in Britz and Rudow. Gropiusstadt is the smallest local district, yet there are nearly as many people living in Gropiusstadt as there are in Buckow, Rudow, or Britz [36]. This makes (North) Neukölln and Gropiusstadt very densely populated, while Britz and Rudow are lightly populated.

Looking at the age distribution, there is a similarly heterogeneous pattern in the age group 27–45 years (Table 2). Nearly half of the population of (North) Neukölln is between 27 and 45 years old. Conversely, looking at the age group 55–65+, (North) Neukölln has the smallest share, whereas this age group often has the largest share of inhabitants in the other local districts. This makes the local districts of Britz, Rudow, Gropiusstadt, and Buckow by far the oldest local districts, with well over one-third of the population 55 years of age and older. On the other hand, the share of children, young adults, and adults younger than 27 years old in all five local districts is more or less the same (ibid.).

#### 2.1.2. Built Structures

**Figure 2:**
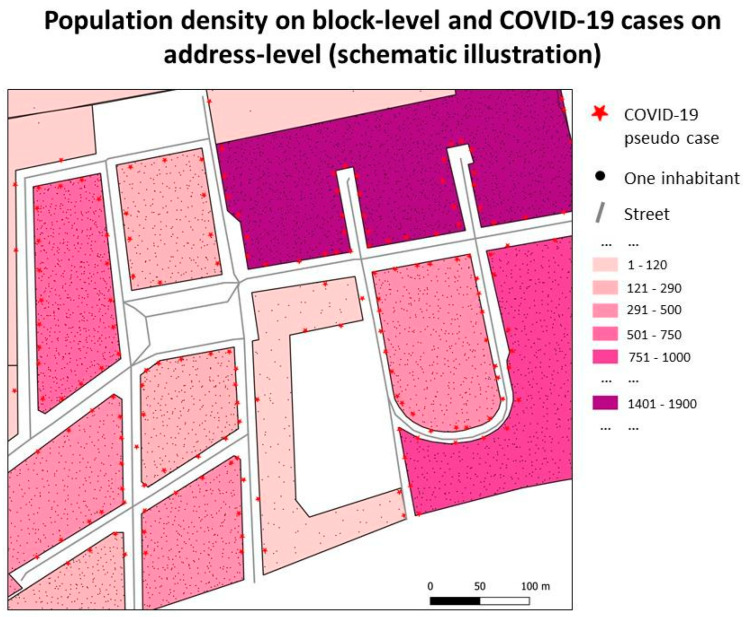
Blocks with their population densities. The points in the block represent the corresponding inhabitants—one for each person living in that block. Please note that even though it looks like one COVID-19 case, there could be five, ten, or fifteen positive COVID-19 cases behind each star. This is because each star represents the (here, pseudo) residential address of a COVID-19 case. If there are several people in the same residential building, they all have the same address, resulting in the same coordinates. Data source: Stadt-Berlin [41]; CRS: 25833.

The built structures in Neukölln can be classified as follows (Figure 2c): (1) ‘dense building structures’ that include (among others) perimeter blocks two to eleven stories high with closed or opened courts; (2) ‘dispersed building structures’, which are town houses, mansions, and detached houses with private gardens; (3) ‘industrial areas’, which are made up of industrial parks or spatial units, where commerce and industry dominate; (4) ‘traffic areas’, which include large parking spots, railyards, etc.; (5) ‘communal and special usage’, which are areas designated for culture, education, administration, religion, etc.; and (6) ‘green areas’, which are parks, forest, water, agriculture, etc. [38].

In (North) Neukölln, dense building structures and industrial areas are predominant. The green areas are concentrated in the west and east, and there are only a few dispersed building structures. Moving further south, the picture changes. Britz has a lot of green areas in the south, west, and east. Its built structure is a mixture of dense building structures stretching from north to south in its centre to Gropiusstadt, dispersed building structures, and industrial areas. Gropiusstadt is an agglomeration of dense building structures with little green area. Buckow and Rudow are dominated by dispersed building structures. Buckow has some dense building structures to its south. Both local districts have few industrial areas. Both districts border large green areas and have green areas within their administrative units to the south.

To summarise, a gradient is observable from (North) Neukölln to Rudow. The further south one is in Berlin-Neukölln, the more dispersed the built structure becomes. Dense building structures are replaced by dispersed building structures, with Gropiusstadt posing an exception as it is characterised by a low to very low socio-economic situation (see Figure 1). With Britz, Berlin-Neukölln has a green centre bordered by Rudow and Buckow, with their mostly dispersed building structures and green areas in the south.

#### 2.1.3. The Socio-Economic Situation

Figure 1d depicts the socio-economic situation in Berlin-Neukölln [39]. The index is calculated using the unemployment rate, child poverty, and people receiving social benefits even though they are not unemployed, thus living below the poverty level. All but one of the planning units classified as very low are located either in (North) Neukölln or Gropiusstadt. Most of the planning units classified as low are also located in (North) Neukölln and Gropiusstadt, with a few located in Britz and Buckow. Despite these few areas being classified as low, the other planning units in Britz and Buckow are all classified as average. Rudow shows a very homogenous picture with an average socio-economic situation. A gradient from north to south in Berlin-Neukölln is observable. The districts south of (North) Neukölln have a better socio-economic situation, with Gropiusstadt posing an exception.

### 2.2. Data

#### 2.2.1. COVID-19 Case Data

For this study, we obtained 105,583 anonymised PCR test-confirmed COVID-19 case data from the local health department of Berlin-Neukölln [42] from 29 February 2020 to 14 April 2022, with the following attributes: age, sex, residential address of tested person (street, house number, zip code), latitude, longitude, and date when the positive PCR test result was added to the local health department’s database. PCR tests were undertaken in specialised testing facilities or at medical facilities (such as public and private doctors’ offices, hospitals, etc.). Inconsistencies in the data were removed, and only cases with a valid address consisting of a street, house number, and zip code were retained. Cases located in nursing homes and refugee shelters were removed to avoid bias in the COVID-19 distribution. They pose special cases in COVID-19 research because the infectious dynamics in these locations follow a different paradigm than in other forms of living due to their organisational structure and living conditions [43,44,45,46]. After data cleaning, 100,908 PCR-confirmed COVID-19 cases (95.57%) on the address level were available for this study. Duplicate data points with the same coordinates, i.e., the same address of a residential unit, were included in the dataset to account for sporadic and clustered cases [26].

#### 2.2.2. Control Data

Population data for Berlin at the small-scale neighbourhood level (statistical blocks) from 2020, available from the Statistical Office Berlin-Brandenburg [41], were used. There are 1082 statistical blocks in Berlin-Neukölln, with a population ranging from 0 to 3273 and an average population size of 303 (see also Figure 1a). The sizes of the blocks vary strongly. The largest block is 45.101 m^2^, while the smallest block is 1.937 m^2^, with an average size of 26.418 m^2^. Most studies developing a spatial-temporal relative risk surface draw samples when it comes to control selection [26,47,48]. However, this involves the danger of oversampling a specific group or spatial unit. Because of that, this study used the whole population of Berlin-Neukölln as controls. This was achieved by sampling n points into the polygons representing the neighbourhoods, where n is the number of people living in the neighbourhood (Figure 2). All population data were included as control data since a differentiation of the population of nursing homes or refugee shelters was not possible.

### 2.3. Methods

#### 2.3.1. The Spatial Relative Risk Function

The spatial relative risk function [49,50] is obtained by comparing the smoothed density of cases and controls within a geographical region [51] using occurrences of a disease, commonly known as cases (here COVID-19), and the underlying population at risk, commonly known as controls. The densities of the cases and controls are calculated using kernel smoothing [52], and the relative risk function, identifying areas of high and low risk, is the calculation of their ratio, with the cases being the ‘numerator’ while the controls are the ‘denominator’ [53,54]. Bithell’s [49,50] original idea of the relative risk function was further developed by Lawson and Williams [55] and Kelsall and Diggle [56] and extended to the spatial setting by Kelsall and Diggle [57]. Since then, kernel smoothing and/or developing spatial relative risk surfaces have been successfully applied in various epidemiological cases [47,48,58,59,60].

#### 2.3.2. Kernel Density Estimation

In most cases, when a spatial relative risk surface of point data is developed, kernel smoothing is used to estimate densities [51]. The density estimation at each location includes the concentration of points within the neighbouring area, which is defined by the bandwidth of the kernel. Because of this, the selection of the appropriate bandwidth is of crucial importance and has received a lot of attention in the scientific community [61,62,63,64,65,66]. Since the human population is dispersed very heterogeneously [65]—and this is especially true for the case of Berlin-Neukölln—a kernel with a fixed bandwidth is not the best choice. When choosing a lot of smoothing (large bandwidth), the kernel will most likely not capture details on a small scale in regions with a lot of data points, while it will probably work well in regions with little data. On the other hand, a fixed-density estimator with little smoothing (small bandwidth) will supposedly work well in regions with a lot of data, while it will presumably produce false-positive areas of high risk in areas with few data points. Thus, developing a spatial relative risk surface with a fixed bandwidth to estimate the densities in an urban setting most likely results in the choice of a bandwidth that is a compromise between a large and a small bandwidth. This is not optimal because important details that might give etiological clues about the dispersion of a disease could be overlooked. That is why an adaptive bandwidth for the kernel estimation of spatial relative risk is needed [51,53,65,67]. It is adaptive in the sense that the smoothing applied through the bandwidth adapts to the spatially varying underlying data, hence its varying density [53,65].

### 2.4. Calculating the Spatial Relative Risk Surface

The analyses in this study were performed in R [68] using the freely available R packages sparr [53] and spatstat [69]. Following Davies et al. [53], Elson et al. [48], and Elson et al. [26], as well as the well-documented sparr package (cf. [70] for the documentation), the raw data were used to create a combined case-control dataset. We divided the data set into five waves (with the fifth wave still ongoing) and two summer plateaus (Table 3) according to the wave classification of the Robert Koch Institute [2]. To calculate the spatial relative risk function, a ‘pilot’ and ‘global’ bandwidth estimation must be provided to initialise the estimator [48]. This is because the spatial relative risk surface was calculated with an adaptive bandwidth. The bandwidths for the ‘pilot’ estimation were calculated separately for cases and controls using bootstrapping [71] with an oversmoothed [72] bandwidth. The ‘global’ bandwidth was calculated on the pooled dataset of cases and controls, again using the oversmoothing principle of Terell (ibid.). With the ‘pilot’ and ‘global’ densities, the relative risk function was calculated as asymmetric adaptive kernel log-relative risk function estimates. Uniform edge correction [53] was applied to avoid bias at the boundary of the (finite) study region [73]. *p*-value surfaces aid in the interpretation of relative risk estimates by providing pointwise measures of statistical significance. The *p*-value surfaces are the result of testing the null hypothesis that risk = 1 at a particular point. The alternative hypothesis states that risk is either <1 or >1 at the same point. The contours superimposed on the risk surfaces correspond to significant values of *p* and help to distinguish areas of high or low risk from noisy artefacts in the estimated relative risk function. Using the z-test recommended by Hazelton and Davies 2009 [74], *p*-value surfaces were estimated for each surface at the 0.01 and 0.001 significance levels, highlighting areas of significantly higher or lower risk by superimposing contour lines denoting p onto each surface [65,67].

## 3. Results

**Figure 3:**
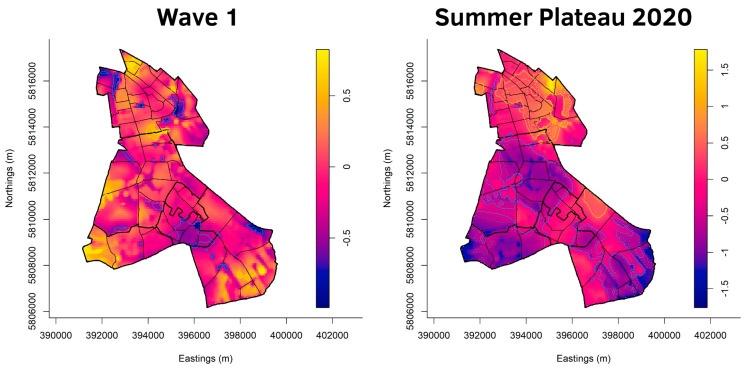
Asymmetric adaptive kernel log-relative risk surface for Berlin-Neukölln with asymptotic *p*-value surfaces at the 0.01 (solid line) and 0.001 (dashed line) significance levels. Yellow lines: Areas of statistically significant high risk; Blue lines: Areas of statistically significant low risk. Scale: log_2_n.

**Figure 4:**
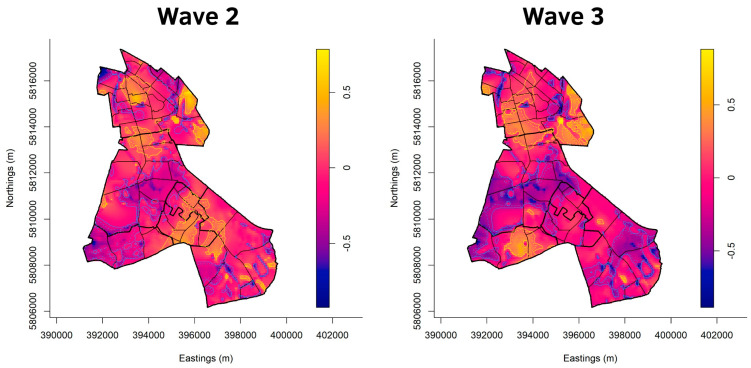
Asymmetric adaptive kernel log-relative risk surface for Berlin-Neukölln with asymptotic *p*-value surfaces at the 0.01 (solid line) and 0.001 (dashed line) significance levels. Yellow lines: Areas of statistically significant high risk; Blue lines: Areas of statistically significant low risk. Scale: log_2_n.

**Figure 5:**
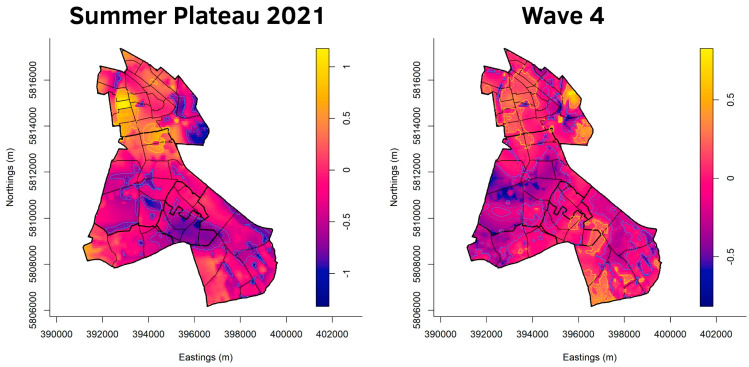
Asymmetric adaptive kernel log-relative risk surface for Berlin-Neukölln with asymptotic *p*-value surfaces at the 0.01 (solid line) and 0.001 (dashed line) significance levels. Yellow lines: Areas of statistically significant high risk; Blue lines: Areas of statistically significant low risk. Scale: log_2_n.

**Figure 6:**
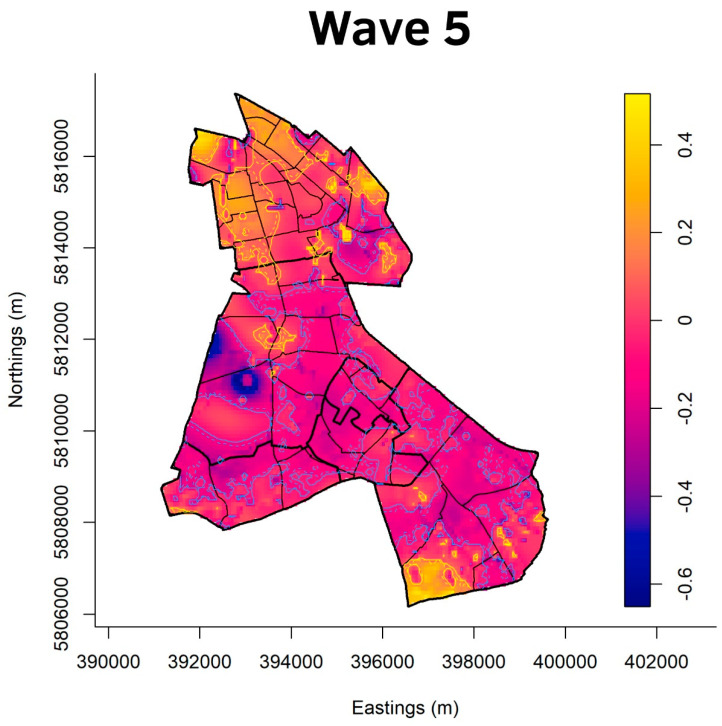
Asymmetric adaptive kernel log-relative risk surface for Berlin-Neukölln with asymptotic *p*-value surfaces at the 0.01 (solid line) and 0.001 (dashed line) significance levels. Yellow lines: Areas of statistically significant high risk; Blue lines: Areas of statistically significant low risk. Scale: log_2_n.

**Figure 7:**
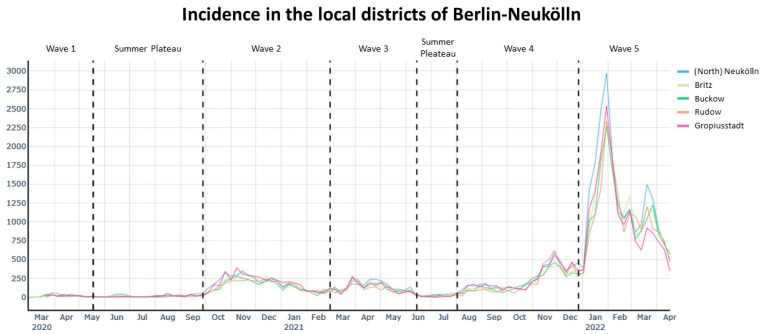
Incidence (cases per 100,000) in the local districts. The dashed lines indicate the waves as defined in Table 3.

Figure 3, Figure 4, Figure 5 and Figure 6 show the cumulative relative risk surface of Berlin-Neukölln for the four waves and two summer plateaus (Table 3), highlighting areas of high risk (red to yellow) and areas of low relative risk (purple to blue). The lines delineate areas of statistically significant high and low relative risk. The scale to the right of the plots indicates an increased- or lower-relative risk, e.g., a 1.5 times higher or 1.5 times lower relative risk compared with other areas that have the value 0. The local districts as well as the planning units are superimposed with black lines. Because the spatial relative risk surface is produced with an adaptive bandwidth, the bandwidth in each figure is different because the bandwidth reflects the case density for each time slice. However, the surfaces are comparable when considering the different scales. Additionally, Figure 7 shows the incidence for the local districts, divided by the waves as defined in Table 3.

In wave one, there are only a few areas of significant high risk (Figure 3). The largest area of statistically high risk is in the north of (North) Neukölln, while other areas are located at the border between (North) Neukölln and Britz and in the east of Britz, and four very small areas are in the very south of Rudow. Buckow is its most southeastern point, an area of statistically significant high risk. The spatial relative risk surface also reveals areas of low risk. They are mostly located in (North) Neukölln and Gropiusstadt. A few patches of statistically significant low risk are scattered over the other districts, but they remain rather small.

The summer plateau 2020 in Figure 3 shows a completely changed picture. The infectious dynamic has nearly completely moved to (North) Neukölln, with a large share of the local district marked as an area of significant high risk. Additionally, considering the scale, the risk in (North) Neukölln is higher than in the first wave. All the other local districts are mostly identified as areas of low risk, with one patch of statistically significant high risk in the north of Rudow. Conversely, the risk is also lower in these areas than in the first wave.

Figure 4 shows the spatial relative risk surface for waves two and three. During the second wave, half of Gropiusstadt showed up as an area of statistically significant high risk. (North) Neukölln has approximately an equal number of areas of significant high and low risk. While in Rudow, there are areas of significant low risk in the south, there is a rather large area of statistically significant high risk bordering Gropiusstadt. Buckow shows a mixed picture, with large areas of statistically significant low risk in its southern part and a few areas of elevated risk in its northwestern part, where it borders Gropiusstadt. Britz has a large area of statistically significant high risk in its north, bordering (North) Neukölln.

The spatial relative risk surface for the third wave (Figure 4) reveals that in Rudow, there are hardly any areas of statistically significant high risk but mostly areas of statistically significant low risk. Gropiusstadt is no longer an area of high risk but is partially an area of low risk. In the south of Buckow, there is a large patch of statistically significant high risk. Britz, to its north, is a large area of low risk. (North) Neukölln became an area of mostly statistically significant high risk, with patches of low risk here and there.

Figure 5 shows the summer plateau of 2021 as well as wave four. During the summer plateau of 2021, the overall area of statistically significant areas of high and low risk decreased in all the local districts. (North) Neukölln is a mixture of areas with high and low risk, with one large area of statistically significant high risk dominating it. Another particularly large patch of elevated high risk is located on the border of Britz and (North) Neukölln, reaching more into Britz than (North) Neukölln. The local districts south of (North) Neukölln have no areas of statistically significant high risk but only areas of low risk. Gropiusstadt is mostly an area of statistically significant low risk.

During the fourth wave (Figure 5), nearly all of Britz is highlighted as an area of statistically significant low risk, as is Buckow. (North) Neukölln shows a mixed relative risk surface, with more areas of significant high risk than areas of low risk. Gropiusstadt has one fraction of elevated high as well as low risk. Rudow shows a mixed picture, with areas of statistically significant high risk in its northeast and southeast as well as areas of statistically significant low risk in the south and southeast.

Figure 6 shows wave five. The areas of statistically significant low risk in all districts become patchier. There are hardly any large areas of contiguous, statistically significant low risk. (North) Neukölln is mostly covered by areas of statistically significant high risk. One part of Britz is at elevated high risk, while there is a rather large area of statistically significant high risk in the southeast of Rudow. Half of Gropiusstadt is identified as an area of statistically significant low risk.

Finally, Figure 7 shows the incidence per 100,000 inhabitants per local district divided into the waves and summer plateaus. By looking at the incidence, the course of the COVID-19 pandemic becomes clear. The further Berlin-Neukölln progressed in the pandemic, the more cases there were, peaking in the Omicron wave with an incidence close to 3000.

## 4. Discussion

Figure 3, Figure 4, Figure 5 and Figure 6 highlight the advantage of point pattern analysis compared with aggregated administrative units of analysis. The areas of statistically significant high and low risk reach over the border of one local district into another. Looking at the smaller statistical units, the planning units, one can clearly see that the infectious dynamic is often only located in parts of them. Yet, with analysis run on aggregational units, such as administrative areas, these local dynamics cannot be detected. Instead, the whole aggregational unit would be marked as an area of high or low risk. For policy makers, point pattern analysis might be of special interest because community actions can be planned more accurately by identifying areas within areas, especially on a small scale.

Additionally, the spatial relative risk surfaces also reveal possible spillover effects. Looking at the northern border of Gropiusstadt, where it borders the western part of Buckow in the second wave, an area of statistically significant high risk extends over Gropiusstadt and parts of Buckow. Another area of statistically significant high risk stretches over the southwest parts of Gropiusstadt and Rudow. There have been initial attempts to describe spatial spillover effects in COVID-19 [75,76], but further research is needed to identify the drivers for these spatial phenomena in Berlin-Neukölln.

### 4.1. Socio-Economic Situation

At the local district resolution level, some interesting insights are gained by producing spatial relative risk surfaces. When it comes to areas of statistically significant high risk, (North) Neukölln seems to be the most affected local district of Berlin-Neukölln throughout all the waves except for the first wave. The relative risk surface reveals areas of relative high risk more often than areas of relative low risk. Additionally, during the summer plateau 2020, it is apparent that all the infectious dynamics are happening in (North) Neukölln, whereas in the rest of Neukölln, the relative risk surface reveals only areas of statistically low risk, except for one area in the north of Rudow. This raises the question of what distinguishes (North) Neukölln so much from all the other districts, especially during the summer plateau of 2020. One possible explanation might be the socio-economic situation of its inhabitants, which is mostly classified as low to very low. The same is true for Britz, where most of the infectious dynamic is taking place in the northern part. The southern part of Britz is often an area of statistically significant low risk. Looking at the socio-economic situation in Britz, the north is classified as low, while the southern planning units are classified as average. Many health science studies suggest a connection between socio-economic factors and health in general (e.g., [77,78]) as well as vulnerability to environmental hazards and socio-economic factors (e.g., [79,80]). This has been shown for COVID-19 as well (e.g., [81,82]). In contrast, seven statistical blocks in the northern part of (North) Neukölln exhibit an average socio-economic situation. However, there are areas of statistically significant high risk located in these blocks. This suggests that the socioeconomic situation alone is not the only driving factor for COVID-19. Since the built structure in these blocks is dense, with little green areas in between, the built structure might be another driving factor for COVID-19. Spatial regression analysis could lead to further insights here.

Another possible insight gained by the spatial relative risk surface is that, during the first wave, the more affluent people were affected. With regard to the spatial relative risk surface of wave one, only the area of statistically high risk located at the border of (North) Neukölln and Britz is in an area with a very low socio-economic situation. The other areas of statistically significant high risk are located in areas with an average socio-economic situation in (North) Neukölln, in the east of Britz and Buckow, and in the south of Rudow. In addition, the colouring of the spatial relative risk surface reveals patterns of elevated risk in more affluent areas. Additionally, areas of a low to very low socio-economic situation, such as Gropiusstadt and parts of (North) Neukölln, are highlighted as areas of statistically low risk. Recent research suggests that more affluent, hyper-mobile people carried COVID-19 into their countries, thus being the driver of the pandemic at its very start [83,84,85]. Another possible explanation might be that this group of people (more affluent and often better educated) was more sensitive to the symptoms at the beginning of the pandemic and got tested more often, leading to more identified cases in this group.

### 4.2. Built Structures

Looking at the built structures, some interesting insights arise. Despite having nearly the same built structure, population density, and socio-economic situation as (North) Neukölln, Gropiusstadt was only an area of statistically significant high risk in the second wave. In wave four, it only had a few statistically significant high-risk areas, whereas throughout all the other waves, it remained an area of low risk, often with large areas of statistically significant low risk. This raises the question of why Gropiusstadt is often an area of statistically low risk, even though it shares so many characteristics with (North) Neukölln. One possible explanation might be the way Gropiusstadt and (North) Neukölln differ in their built structures, even though Figure 1c proposes similar characteristics between the two local districts. A closer look is necessary here. Gropiusstadt was built in 1962–1975 by the Bauhaus architect Walter Gropius in accordance with the Athens Charter as a satellite town with 19,000 residential units and up to 30-storey-high buildings [86]. In (North) Neukölln, there are mostly houses built in the Wilhelminian style that are 4–5 stories high. In high-rise apartment complexes, such as Gropiusstadt, social isolation is experienced throughout all age groups, but especially in the age groups of students and those 60 and older [87]. Additionally, the number of places for social interaction, such as bars or cafes, is very limited, offering hardly any places for interaction between the residents in Gropiusstadt. This is very different in (North) Neukölln, a local district known for its shops, bars, cafes, and restaurants, additionally making it an attraction for tourists. Combining the low number of places for social interaction in Gropiusstadt with the social isolation that is especially experienced by residents who are 65 and older in high-rise apartment complexes (ibid.), an age group that is dominant in Gropiusstadt, might explain why the relative risk in Gropiusstadt is low throughout nearly the whole of the pandemic. Further analysis is needed to identify how Gropiusstadt differs from (North) Neukölln here, which finally leads to lower infection rates.

The spatial relative risk surface suggests something else. The built environment in Britz is a mix of dense and dispersed structures, with very large green areas in between. Consider the eastern part of Buckow, which generally remained an area of statistically significant low risk. Only during the third wave did an area of statistically significant high risk appear. The same is true for the western part of Buckow. An area of significant high risk was observed only during wave two. The northern border of the eastern part of Buckow extends along a large green area, while the northern border of the western part of Buckow also extends along a large green area. Moreover, in these parts of Buckow, there are mostly areas of statistically significant low risk. These insights revealed by the spatial relative risk surface suggest that proximity to green areas is beneficial in preventing the spread of COVID-19. People can meet outside rather than inside. The impact of green spaces on people’s mental and physical health has been subject to research for a while now (see, for example, [88] and the literature cited there). However, how proximity to green spaces affects the infectious dynamics of COVID-19 has not yet been fully researched. Most of the research focuses on the general benefits of urban green spaces during the COVID-19 pandemic [88,89,90,91] but does not focus on the possible connection between proximity to urban green spaces and infection events.

Consider Rudow, where most of the area consists of buildings classified as dispersed structures. This appears to have a beneficial impact on the number of infections, which seems plausible. Having a garden leads people to stay home; if you meet people, you can do this in your garden instead of inside, and so forth. Furthermore, the population density in Rudow is, compared with (North) Neukölln or Gropiusstadt, for example, rather low. This reduces the number of people you live with, making it easier to isolate a person who tests positive for COVID-19, thus leading to fewer settings where COVID-19 can be transmitted. The low relative risk in Rudow might also be a combination of the socio-economic situation (which is mostly classified as middle), the built structure, and population density. First studies have already investigated the relation between built structure and COVID-19 [92]. A well-designed regression analysis might be able to dissect the factors that lead to a lower relative risk in Rudow.

### 4.3. Limitations

Looking at the spatial relative risk surfaces produced, one has to keep in mind that this method is purely exploratory to help identify risk factors and underlying processes that may be addressed in a suitable next step, e.g., spatial regression analysis.

There is a bias in the case data because these are most likely not all the COVID-19 cases in Berlin-Neukölln due to limitations in testing capacities and the willingness of each individual to get a PCR test if COVID-19 symptoms develop. This is especially true for Omicron, which (a) exceeds the PCR testing capacities because of its high infectious dynamics [93] and (b) often has a mild course of infection compared with other variants such as Delta [94], which might lead to a lower incentive to get tested. On 5 February 2022, the city of Berlin changed the testing strategy to preserve PCR test capacities: a properly performed positive COVID-19 rapid antigen test during times of high incidence makes a follow-up PCR test unnecessary. Citizens are obligated to treat the positive antigen rapid test like a positive PCR test and isolate themselves. This excludes persons working in hospitals, doctors’ offices, care facilities, and facilities for integration assistance. They still had to take a PCR test when they had a positive antigen rapid test result [95]. This means that the PCR-based COVID-19 case data used in this study does not reflect the COVID-19 case numbers completely.

Furthermore, the date assigned to each COVID-19 case is the date when the case was added to the database. This date differs from the date when the specimen was taken. This is especially true during times of high incidence because the capacities of the laboratories were exceeded and tests were performed before weekends and public holidays. Laboratories and health departments do not work on public holidays or on the weekend, and because of that, there is also a time lag between the date when the specimen was taken and the date when the local health department was informed about a positive COVID-19 infection. On top of that, there are eleven cases where people have been tested a number of times within a time frame of 20 days. These cases were included in the analysis.

Additionally, one has to keep in mind that the case numbers might be biased by the availability of testing facilities. The distribution of the registered 91 test centres (on 24 February 2021) in Berlin-Neukölln was not homogenous throughout the pandemic. However, tests were also available at doctors’ offices and in other facilities throughout the pandemic. Moreover, people probably did not necessarily choose the closest testing facility to their place of residence to get a COVID-19 test, but the choice might have been affected by other factors such as proximity to a commuter route or other factors. Even though we were able to exclude cases located in nursing homes and refugee shelters, this was not possible for the control data as information about the number of residents was not available. Finally, we excluded duplicates where the same case ID was recorded (*N* = 2), whereas duplicates for the same person were included in this analysis (*N* = 11 for positively recorded PCR tests within a period of 20 days).

While we are aware of the limitations of the data and applied methods, our study presents new findings for the initial exploration of the COVID-19 cases as a starting point for local policies and future studies on underlying factors. We were able to verify our findings with experts from the local health department to address the gap in information and knowledge on the spatio-temporal dynamics of COVID-19 throughout the pandemic. Even though decisions such as a lockdown are made on the national level, federal, state, and municipal policies play an important role in not only local data assessment and analysis but also in policy implementation. In the case of Berlin, the district level, for example, is a decisive one for policy-decision-making. Several district-level measures, such as the planning of local vaccination initiatives or locally adapted information campaigns, can clearly benefit from more detailed knowledge.

## 5. Conclusions

To our best knowledge, this is the first exploration of the spatio-temporal distribution of COVID-19 in Germany using address-level data, describing areas of statistically high and low risk on a small scale, unbiased by administrative boundaries. The data quality for our study is different since it uses all PCR-positive tested results (100,908) that are located in the district of Berlin-Neukölln across the whole duration of the pandemic as well as the entire population data on the small block level and not on a large grid like other health science studies often use. Additionally, no sample of the population was drawn, which overcomes the danger of oversampling a specific area or group. A major strength of our study is that by using all PCR test results (not just those that were conducted when a patient sought medical attention), we can reduce bias towards cases with a severe course.

By using point pattern analysis, this study is able to give detailed insight into the spatio-temporal dynamics of COVID-19 in Berlin-Neukölln. By highlighting areas of statistically significant high and low risk, the continuous spatial relative risk surfaces give important etiological clues, unbiased by aggregational units such as administrative boundaries. Exploring COVID-19 in Berlin-Neukölln through a spatial relative risk surface highlights areas for further research, namely, is there a meaningful connection between proximity to urban green spaces and infection events? Why is Gropiusstadt not as affected by COVID-19 as (North) Neukölln, even though these two local districts have a lot in common? Is there evidence that the more affluent and mobile people were the drivers of the pandemic during the first wave and later resided in ‘safe’ areas? How important are built structures as drivers of COVID-19? We suggest that small-scale regression analysis might be able to answer these questions.

Finally, developing a spatial relative risk surface is very straightforward, which means that it can be applied by policy makers on a daily basis. By overcoming the MAUP and identifying areas of high (and low) infection within areas, policy makers can target areas of high infection rates with high precision. This makes the spatial relative risk surface one of the basic tools to properly plan for community actions and to protect vulnerable groups from infection with targeted community actions, such as offering free tests, inoculation and information campaigns, and so forth. However, this requires the assessment and analysis of detailed data. When it comes to health data, these data contain sensitive personal information. Yet, privacy regulations should make cooperation between scientists and health departments possible to enable both researchers and public stakeholders to harness the hidden etiological clues in health data. However, one must keep in mind that the calculation of a spatial relative risk surface is computationally demanding. It can only be performed with the appropriate hardware. Large datasets are especially challenging when it comes to point pattern analysis. This is where aggregated data may have advantages.

Perhaps the most important conclusion of this article is that there is a strong need for developing adequate workflows and providing resources for data assessment and analysis in health departments.

## Figures and Tables

**Table 1:** Population characteristics in Berlin-Neukölln [36].

Administrative Unit	Area (km^2^)	Total Population	Relative Share	Population Density (per km^2^)
(North) Neukölln	11.7	164.636	50.2%	14.071
Britz	12.39	42.846	13.06%	3.458
Rudow	11.82	42.631	13%	3.607
Buckow	6.34	40.146	12.24%	6.332
Gropiusstadt	2.66	37.686	11.49%	14.168
Total	44.91	327.945	100%	Ø 7.283

**Table 2:** Population of the district of Berlin-Neukölln grouped by age and its local districts in 2020 [36]. The first number is the total population of this age group in the district, and the second number is the relative share.

Administrative Unit	Age in Years							
	Under 6	6–15	15–18	18–27	27–45	45–55	55–65	65 and Older
(North) Neukölln	9.726	12.636	3.713	16.930	68.061	19.604	16.693	17.273
	5.91%	7.68%	2.26%	10.28%	41.34%	11.91%	10.14%	10.49%
Britz	2.498	3.449	1.011	4.278	11.129	5.490	6.165	8.826
	5.83%	8.05%	2.34%	9.98%	25.97%	12.81%	14.39%	20.6%
Rudow	2.454	3.477	1.222	3.779	8.197	5.824	6.619	11.059
	5.76%	8.16%	2.87%	8.66%	19.23%	13.66%	15.53%	25.94%
Buckow	2.267	3.234	1.043	3.633	8.229	5.236	5.758	10.746
	5.65%	8.06%	2.6%	9.05%	20.5%	13.04%	14.34%	26.77%
Gropiusstadt	2.503	3.021	990	3.598	8.456	4.247	4.889	9.973
	6.64%	8.02%	2.62%	9.55%	22.44%	11.27%	12.98%	26.46%
Total	19.448	25.817	7.979	32.218	104.081	40.401	40.124	57.877
	5.93%	7.87%	2.43%	9.82%	31.74%	12.32%	12.23%	17.65%

**Table 3:** Time spans of waves and their variants of concern (VOC) as defined by the RKI [2].

	1. Wave	Summer Plateau	2. Wave	3. Wave	Summer Plateau	4. Wave	5. Wave
Date	02 March 2020–17 May 2020	18 May 2020–27 Septemnber 2020	28 September 2020–28 February 2021	01 March 2021–13 June 2021	14 June 2021–01 August 2021	02 August 2021–26 December 2021	27 December 2021
VOC	-	-	-	Alpha	-	Delta	Omicron

## Data Availability

The data that support the findings of this study were obtained from the local health department in Berlin-Neukölln, but restrictions apply to the availability of these data, which were used solely for the current study and are therefore not publicly available. Data are available from the authors upon reasonable request and only with the permission of the local health department in Berlin-Neukölln.
